# Twisting loop technique for anterior mitral cords shortening and evolution

**DOI:** 10.1186/1749-8090-8-S1-O279

**Published:** 2013-09-11

**Authors:** I Moriones, JL Fernandez, R Sanchez, L Jimenez, R Sadaba, F Gomez

**Affiliations:** 1 Department of Cardiac Surgery, CHN, Pamplona, Spain

## Background

It is a known fact that the surgery of the anterior leaflet presents more difficulties and more dubious results than in the posterior leaflet. The use of artificial chordae is not always effective and measure its length is a challenge for the surgeon. So we present our long-term results with our new original technique for shortening the elongated chordae we call,” twisting loop technique “(TLT).

## Methods

This technique is made by twisting each prolapsed chord, near the edge of the corresponding leaflet, making a loop and tying it to the edge of the leaflet .We analize results in 20 patients .14 were men and 6 women. Mean age was 66+/- 9 years EF =0.55+/-0.10 NYHA CLASS= 2.5+/- 0.7. Mitral regurgitation was severe in all patients This method was applied to 34 elongated chordeae (28 in anterior leaflet and concomitantly in other 6 in posterior leaflet) Etiology was degenerative in all cases Concomitant surgery was: Eight quadrangular resection in P2, Three cleft closure, two A2 plicatures, three aortic prosthesis and three tricuspid rings .In all cases mitral ring was implanted. Mean cross- clamp time was 92+/-21 minutes.

## Results

There was no surgical mortality. Twenty valves where competent at hospital discharge. Mean follow up is 33+/-14 months (range 54-12) Mean postoperative NYHA CLASS was 1.22+/-04 In echocardiographic evolution ,one year later, 19 patients presented mitral competent valves (95%) One case had mild echocardiographic regurgitation. EF =0.56 +/-0.10. Ventricular diastolic diameters decreased from 60+/-9 to 50.5+/-5 millimeters ((p<0.01). . All patients are in good clinical situation.

## Conclusions

Our “twisting loop technique” (TLT) for anterior leaflet demonstrated very good results initially and in long term follow up. Simplicity of this technique is useful to correct mitral prolapsed valves and is our preferred option for these patients. These results must be confirmed with longer follow up studies.

